# Recent Advances in Lateral Flow Assays for Viral Protein Detection with Nanomaterial-Based Optical Sensors

**DOI:** 10.3390/bios14040197

**Published:** 2024-04-17

**Authors:** Min Jung Kim, Izzati Haizan, Min Ju Ahn, Dong-Hyeok Park, Jin-Ha Choi

**Affiliations:** 1 School of Chemical Engineering, Clean Energy Research Center, Jeonbuk National University, 567 Baekje-daero, Deokjin-gu, Jeonju-si 54896, Jeollabuk-do, Republic of Korea; minjeong413@jbnu.ac.kr (M.J.K.); 201610776@jbnu.ac.kr (D.-H.P.); 2 Department of Bioprocess Engineering, Jeonbuk National University, 567 Baekje-daero, Deokjin-gu, Jeonju-si 54896, Jeollabuk-do, Republic of Korea; izzatihaizan22@jbnu.ac.kr; 3 Department of Biotechnology, Jeonbuk National University, 79 Gobongro, Iksan-si 54596, Jeollabuk-do, Republic of Korea; ahnminju@jbnu.ac.kr

**Keywords:** lateral flow assay (LFA), optical sensing, nanomaterial, virus detection, viral protein

## Abstract

Controlling the progression of contagious diseases is crucial for public health management, emphasizing the importance of early viral infection diagnosis. In response, lateral flow assays (LFAs) have been successfully utilized in point-of-care (POC) testing, emerging as a viable alternative to more traditional diagnostic methods. Recent advancements in virus detection have primarily leveraged methods such as reverse transcription–polymerase chain reaction (RT-PCR), reverse transcription–loop-mediated isothermal amplification (RT-LAMP), and the enzyme-linked immunosorbent assay (ELISA). Despite their proven effectiveness, these conventional techniques are often expensive, require specialized expertise, and consume a significant amount of time. In contrast, LFAs utilize nanomaterial-based optical sensing technologies, including colorimetric, fluorescence, and surface-enhanced Raman scattering (SERS), offering quick, straightforward analyses with minimal training and infrastructure requirements for detecting viral proteins in biological samples. This review describes the composition and mechanism of and recent advancements in LFAs for viral protein detection, categorizing them into colorimetric, fluorescent, and SERS-based techniques. Despite significant progress, developing a simple, stable, highly sensitive, and selective LFA system remains a formidable challenge. Nevertheless, an advanced LFA system promises not only to enhance clinical diagnostics but also to extend its utility to environmental monitoring and beyond, demonstrating its potential to revolutionize both healthcare and environmental safety.

## 1. Introduction

Viruses consist of a structure with a genome inside a protein-based shell that reproduces inside hosts. Scientists have estimated that there are approximately 10^31^ viruses living on Earth [1,2]. The replication of the viral genome occurs only inside living cells (hosts), and the hosts can be any of the major cell organisms (prokaryotes, eukaryotes) [2]. Viral infectious diseases are caused by several factors, including evolutionary and adaptive genetic mutations of viruses, population growth and urbanization, population movement, global warming, and climate change, which can affect human life in many ways [3,4,5,6]. For instance, viral infectious diseases include influenza [7,8], severe acute respiratory syndrome (SARS) [9], middle east respiratory syndrome (MERS) [10,11], coronavirus disease (COVID-19) [7,12,13,14], acquired immune deficiency syndrome (AIDS) [15], Ebola virus disease [16], norovirus gastroenteritis [17], and Zika virus infection [18]. For example, the 1918 influenza pandemic was an extremely lethal influenza pandemic caused by the H1N1 virus. It was one of the most widespread and deadly flus witnessed by world economies, and hampered not only lives, but also society and trade-related activities. Due to this incident, approximately 500 million people were infected by influenza, wherein one third of the world’s population suffered at the time [19]. In addition, in the past few years, more than 115 million cases of infection have been confirmed worldwide since the emergence of SARS-CoV-2, and more than 2.5 million deaths have been recorded [8]. As such, since the transmission of the virus has a fatal effect on human life, an effective diagnostic method is needed to control the transmission of the virus.

In general, nucleic acid detection is performed using polymerase chain reaction (PCR) and loop-mediated isothermal amplification (LAMP). For many years, these conventional diagnostic methods have been utilized especially for virus detection. In detail, RNA virus detection techniques include reverse transcription–polymerase chain reaction (RT-PCR), reverse transcription–loop-mediated isothermal amplification (RT-LAMP), and the enzyme-linked immunosorbent assay (ELISA) [20,21,22,23,24]. For instance, diagnosis by real-time RT-PCR using upper respiratory tract samples is the gold standard for virus detection due to its high sensitivity and specificity [22,25,26,27,28]. On the other hand, RT-LAMP can be performed in real time by measuring turbidity or fluorescence using dyes. Real-time RT-LAMP diagnostic tests are simple and sensitive because they only require heating and visual inspection [20,29,30,31]. Meanwhile, in addition to nucleic acid detection, viral protein detection for virus disease detection is gaining attention due to its simplicity. This is because a single gene can express multiple proteins with various biological functions, and proteins expressed by genes can undergo various post-translational modifications. On the other hand, proteins are the final form of gene products and are directly related to biological functions. Therefore, protein biomarkers are effective for early diagnosis [32]. ELISA is a microwell plate-based assay technique that detects and quantifies proteins (antibodies, hormones, etc.). The test can be performed both qualitatively and quantitatively, and generally requires 1 to 5 h of detection [20,33,34,35,36,37]. In the case of sandwich ELISA, the sensitivity is very high [33]. These conventional techniques have contributed significantly to the diagnosis of several viral diseases. However, the disadvantages of being expensive, requiring skilled professionals, and requiring considerable time for the detection process have necessitated the development of affordable, accessible, and rapid viral diagnostic methods.

In line with these needs, the lateral flow assay (LFA), a paper-based technology, has emerged as an alternative for filling voids. LFAs have been successfully utilized in point-of-care (POC) diagnostic applications. It has been reported that the quantitative estimation of LFA results can significantly improve the efficiency of human and veterinary use, biological safety, consumer protection, and ecological monitoring [38]. With the advantages of being affordable, having a simple system with minimal training or infrastructure, having low temperature sensitivity, and having a short diagnostic time [20], this diagnostic platform has excellent advantages in developing and remote countries. This is due to its efficiency, especially under conditions of limited resources and scarce specially trained personnel [39,40]. Another great advantage of LFA-based testing is that it can be performed on various biological samples, including plasma, sweat, saliva, serum, urine, and whole blood. Moreover, the number of samples required for detection is much less than what is required for other conventional analyses [41]. Protein biomarkers such as virus antibodies, antigens, proteases, and surface-presenting proteins can also be detected without sample pretreatment in biological samples, such as respiratory samples taken directly from the nasopharynx, oropharynx, or saliva [42,43,44,45]. Optical sensing methods, such as colorimetric, fluorescence, and surface-enhanced Raman scattering (SERS), have a simple analysis process, high sensitivity, and selectivity, and can deliver analysis information in a convenient and inexpensive way [46]. In recent years, optical sensors using nanomaterials have been actively developed to quickly, sensitively, and specifically identify targets. Nanomaterials also have many useful properties relative to volume, such as a large surface area, a small size, a quantum confinement effect, high surface reactivity, and improved magnetic/electrical/optical properties, which can be appropriately adjusted and applied to optical sensors [47,48,49,50]. In this review, we summarize the components, structures, and principles of LFAs and present recent advances in LFAs for detecting viral proteins by dividing methods used from 2019 to 2024 into colorimetric, fluorescence, and SERS methods.

## 2. LFA Components, Structures, and Principles

**Figure 1:**
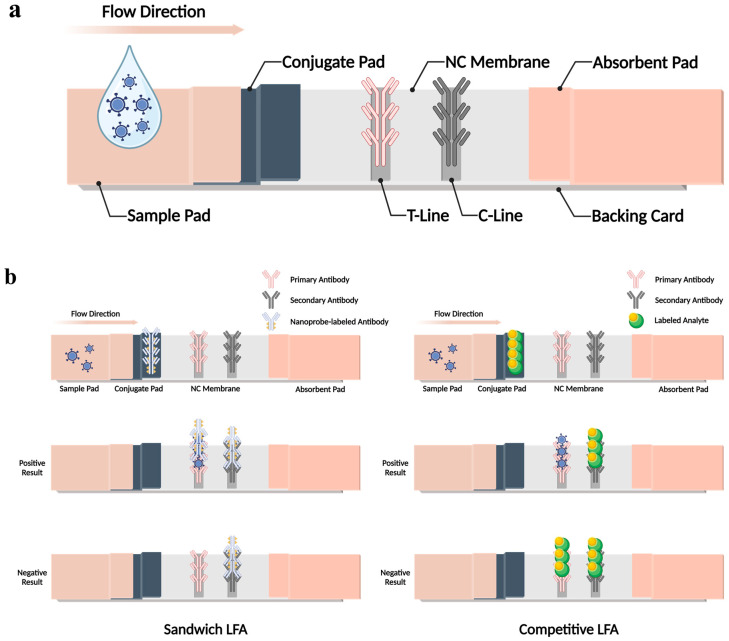
Components and structure of the LFA biosensor for viral protein detection. (**a**) Typical design of LFA strips with their components. (**b**) Widely utilized formats of LFA biosensors. Recreated with permission from [56]. Illustration designed with Biorender.com accessed on 10 March 2024.

Generally, most LFA platforms operate on a basic working principle. The components of the LFA strip include a sample pad, a conjugate pad, a nitrocellulose (NC) membrane, and an absorbent pad that are layered together on top of a backing card (Figure 1a). LFA systems work mainly by adapting the capillary action flow rate concept in which samples containing targets flow from the sample pad along the strip to the absorbent pad. Furthermore, the role of each component in the LFA structures, its principle, and the practiced LFA formats for viral protein detection will be discussed.

The first component of LFA platforms is the sample pad. The sample pad can release analytes with high efficiency to ensure that the sample is compatible for use in subsequent inspection steps. Samples need to be processed beforehand by filtering particulates and changing their pH. This is due to the active binding of sample components and the destruction of substrate components such as mucins, which may interfere with the analysis. Some examples of sample pad materials include cellulose, glass fibers, rayon, and other filtration media. Next, the role of conjugate pads is to accommodate the target complex, keep it stable within its shelf life, and release it efficiently and reproducibly when the analysis is carried out. In detail, to ensure the optimal release and stability of the materials, the conjugate pad is pretreated beforehand through immersion in an aqueous solution of proteins, surfactants, and polymers, followed by a drying process. The materials for the conjugate pad can be glass fibers, polyester, or rayon.

Additionally, NC membranes play a crucial role in LFA platforms. The NC membrane houses two important analysis lines: a test line (T-line) and a control line (C-line). Moreover, NC membranes ensure that the target complexes flow consistently through the two lines, allowing reactions to occur and ensuring that the excess fluid, labels, and reactants escape without binding. Finally, absorbent pads are important for attracting and holding all fluids in this designated area throughout the duration of the analysis. Absorbent pads are as important as other components because, in the presence of fluid flowing opposite to the flow direction, the probability of achieving false positive results is high. Thus, for absorbent pads, high-density cellulose is generally used [51,52].

Moving forward, an in-depth working principle of the LFA platform will be explained. On the LFA strip, when a liquid sample is dropped on the sample pad, the sample will first move through the strip to the conjugate pad. Basically, on the conjugate pad, specifically tagged capture antigens or antibodies and nanoprobes such as gold nanoparticles (Au NPs), fluorophores, and quantum dots (QDs) are present. Hence, in the presence of a target in the samples, nanoprobe-detecting antigen/antibody complexes will form. After the interaction between the targets and nanoprobes, the resulting complex flows to the NC membrane, in which specific antibodies are pre-immobilized on both the T- and C-lines. On the T-line, in the presence of a target, preformed complexes on the conjugate pad will be captured by the primary immobilized antibody. Subsequently, unbound complexes from the T-line flow to the C-line and are captured by the secondary immobilized antibody, indicating that the sample solution has moved sufficiently. Finally, the absorbent pad absorbs the processed and excess samples, preventing them from flowing back to the NC membrane and conjugate pad [39,40,53,54,55].

In the last part of this section, the universally practiced LFA formats for viral protein detection will be introduced. Accordingly, in LFA systems, sandwich and competitive formats are typically employed (Figure 1b). Generally, the sandwich format is used to test large analytes with multiple antigenic sites, and the competitive form is applied for small-scale analyte detection, especially for analytes with low molecular weights and single antigenic determinants. In addition, the main difference between the two formats is the number of antibodies used in each LFA system. Three different types of antibodies for sandwich and two types of antibodies for competitive formats of LFA platforms are used. First and foremost, in the sandwich formation, the three antibodies used are the nanoprobe-labeled antibody (conjugated pad), primary antibody (T-line), and secondary antibody (C-line). In the presence of a target, the nanoprobe-labeled antibody and target will form a complex on the conjugate pad. Then, the formed complex will be recognized and captured by the pre-immobilized primary antibody on the T-line, resulting in sandwich formation. Moving forward, the unbounded nanoprobe-labeled antibody will interact with the secondary antibody, resulting in an observable C-line indicating a positive result. Subsequently, the number of analytes present in the sample can be determined by the color intensity observed on the T-line. In the event of a negative result, only an observable C-line will be observed, indicating that the nanoprobe-labeled antibody interacts with the secondary antibody on the C-line. In the case of the competitive formation of the LFA system, the two antibodies used are primary (T-line) and secondary antibodies (C-line). In the event of the target’s presence in the sample, the labeled analyte will compete with the target to bind with the primary antibody. Consequently, the target will bind to the primary antibody on the T-line, forming a visible red signal on the T-line. In contrast, the labeled analyte will bind to the secondary antibody on the C-line forming a visible red signal on the C-line. Hence, a positive result is obtained. Contradictorily, under the absence of the target, the labeled analyte will bind to both the T- and C-lines, resulting in two visible red lines, hence, portraying a negative result [56,57].

In terms of nanomaterial-based LFA, the general principles are attained with only the addition of nanomaterials integrated in the system. In detail, various nanomaterials are used to aid in detection, especially for the signal amplification of optical sensing. The most commonly used nanomaterials are Au NPs. Au NPs can be synthesized in various sizes and shapes and produce a strong red color when viewed with the naked eye. Carbon-based materials, such as carbon nanoparticles, carbon nanotubes, etc., have been proposed as alternatives to Au NPs. Although this has a weak signal compared to Au NPs, it is stable and has significant cost advantages. In addition to this, dye-containing latex beads are inexpensive and resistant to chemical and physical damage. These nanomaterials also produce a weaker signal than Au NPs. Typically, QDs are the most commonly used in fluorescence-based LFAs and produce strong fluorescence signals through UV radiation. However, QDs have the disadvantages of being toxic and expensive. Upconversion nanoparticles (UCNPs) produce strong fluorescence signals in the near-infrared region; however, UCNPs are expensive and require NIR lasers. Liposomes are also used as nanomaterials for optical sensing. Liposomes are easy to use because they can encapsulate various nanomaterials, but they have the disadvantage of low stability due to their sensitivity to pH and ion strength [58]. In the subsequent chapter, we will present the latest studies on nanomaterial-based LFAs divided into three categories of optical sensing techniques: colorimetric, fluorescent, and SERS.

## 3. Colorimetric-Based LFAs for Viral Protein Detection

Au NPs are plasmon NPs commonly utilized as colorimetric diagnostic probes. Leveraging their inherent optical localized surface plasmon resonance (LSPR) and biocompatible properties, Au NPs are usually integrated into LFA systems. In detail, Au NPs are absorbed and scattered in the visible region, and they absorb millions of times more light than organic dye molecules due to their very high extinction coefficient (~10^9^ for 20 nm Au NPs) [59,60]. Thus, the conjugation of Au NPs with antibodies is a simple, fast, and reliable method for virus detection in the presence of a target virus on the T-line of an LFA system [59,60,61]. 

**Figure 2:**
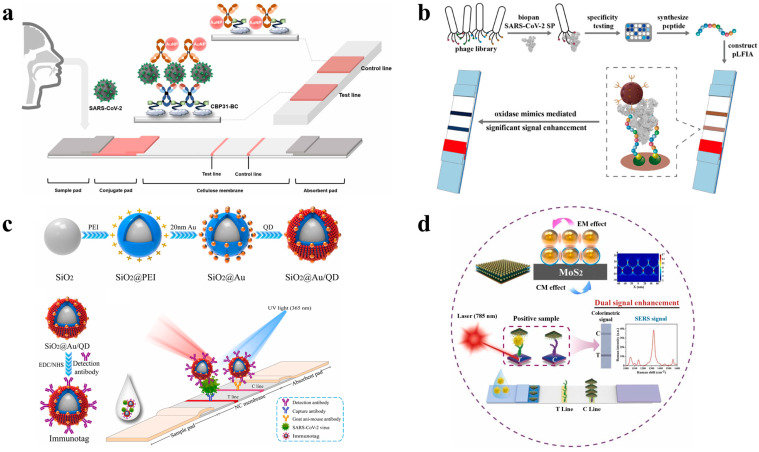
Examples of colorimetric-based LFAs for viral protein detection. (**a**) Scheme of the Au NP-based LFA for the detection of SARS-CoV-2. Reprinted in part with permission from ref. [64]. Copyright 2020 American Chemical Society. (**b**) Scheme of the polydopamine (PDA)@MnO_2_ nanocomposite-based LFA for the detection of SARS-CoV-2. Reprinted in part with permission from ref. [69]. Copyright 2023 American Chemical Society. (**c**) Scheme of the SiO_2_@Au/QD fluorescent labels of a dual-functional LFA for detecting SARS-CoV-2. Reprinted in part with permission from ref. [70]. Copyright 2022 Elsevier. (**d**) Scheme of the MoS_2_@Au–Au-based LFA for the detection of the monkeypox virus (MPXV) antigen. Reprinted in part with permission from ref. [71]. Copyright 2023 Elsevier.

For this reason, many studies of colorimetric-based LFAs have reported the detection of viruses using Au NPs as nanoprobes [62,63,64,65,66,67,68]. For example, Shen et al. reported on a signal-enhanced LFA based on double Au NPs to determine hepatitis B surface antigens (HBsAgs). The Au NPs were modified with biotin and antibodies, Au NPs modified with streptavidin were used as conjugates, and the Au NPs were aggregated twice to improve the signal. When the sample solution flowed along the strip, the first conjugate was captured on the T-line by an antigen–antibody reaction, and the second conjugate was also immobilized on the T-line via biotin–streptavidin, resulting in a detection limit of 0.06 ng/mL HBsAgs [62]. To achieve the rapid diagnosis and field detection of IgM antibodies against the SARS-CoV-2 virus, Huang et al. coated SARS-CoV-2 nucleoprotein on an alkaline membrane, conjugated anti-human IgM with Au NPs to form a detection reporter, and constructed a colloidal Au NP-based LFA. Compared with real-time PCR, the sensitivity and specificity of Au NP-based LFAs are 100% and 93.3%, respectively (Figure 2a) [64]. Cavalera et al. designed a double-line LFA, which consists of recombinant SARS-CoV-2 nucleocapsid (N) protein and Au NPs as colorimetric signal reporters, and functions as a detector by indiscriminately binding to human Immunoglobulin G (IgG), Immunoglobulin M (IgM), and Immunoglobulin A (IgA). Both test lines comprised staphylococcal protein A (SpA) (T-line 1) and N antigen (T-line 2) [66]. The plasmon color-preserved (PLASCOP) Au NP clusters developed by Oh et al. were made by mixing a streptavidin-coated Au NP core with satellite Au NPs coated with biotinylated antibodies. The biotinylated antibody–streptavidin linker forms a gap of more than 15 nm to avoid plasmon bonding between the Au NPs, thus retaining the plasmon color while increasing the overall light absorption. LFA detection with PLASCOP Au NP clusters composed of 40 nm Au NPs had a limit of detection (LoD) of 0.038 ng/mL, indicating high detection sensitivity for the SARS-CoV-2 N protein [67].

Enzyme signaling enhancement has traditionally been used for ELISA and colorimetric LFAs. Peroxidase enzymes have the advantages of being commercially available, stable, and inexpensive. The native peroxidase enzymes used in ELISA and other types of immunoassays are mainly horseradish peroxidase (HRP) and alkaline phosphatase (ALP) [72]. HRP is preferred for conjugation with LFA nanoprobes because it is stable, cost-effective, and easy to form into HRP-labeled antibodies/antigens for immunological recognition compared to other enzymes [73,74]. For example, an LFA platform has been proposed that can further amplify colorimetric signals by adding HRP to Fe_3_O_4_/Au core–shell magnetic NPs. The developed method detected 400 PFU/mL of SARS-CoV-2 in phosphate-buffered saline (PBS) buffer and detected 1200 PFU/mL of SARS-CoV-2 in saliva samples [75]. In addition, LFAs have also been proposed using a combination of Au NPs and ALPs for the high-sensitivity detection of virus X (PVX). Compared with natural enzymes, nanozymes have advantages such as low cost, high stability, and durability compared to natural enzymes [76]. For this reason, substances exhibiting peroxidase-mimicking activity (PMA) are sometimes employed as nanoprobes in LFAs [77,78]. Panferov et al. used Au@Pt core–shell NPs with PMA as probes, reducing the detection limits of PVX in tuber and leaf extracts to 4 and 8 pg/mL, respectively [79]. Sun et al. prepared LFAs enhanced by Au@Pd@Pt core–shell NPs to detect SARS-CoV-2 N proteins. Because Au@Pd@Pt nanozymes have good peroxidase-like activity due to their dendrite morphology and uniform particle size, they can generate catalytic signals even in small amounts [80]. Dong et al. used PDA@MnO_2_ nanocomposites with PMA as colorimetric labels, lowering the detection limit of SARS-CoV-2 spike antigens to 8.01 pg/mL (Figure 2b) [69].

Recently, dual detection methods have been developed to compensate for the sensitivity of colorimetric-based LFAs. Han et al. proposed a colorimetric and fluorescent dual-functional LFA that detects the spike 1 protein of SARS-CoV-2. For strong colorimetric and fluorescent signal generation, single dispersion, and high stability, a single-layer shell was formed by mixing 20 nm Au NPs with a SiO_2_ core with QDs to generate a single-layer shell, resulting in a strong colorimetric and fluorescent signal and ensuring good monodispersity and high stability, thus creating a novel bifunctional immune marker. The colorimetric concentration of LoD is 1 ng/mL, and the fluorescence is 0.033 ng/mL (Figure 2c) [70]. Cheng et al. built a platform to simultaneously detect common respiratory viral influenza A and respiratory bacteria by forming a multi-layered double-signal nanofilm on a 16 nm layer, two layers of colorimetric Au NPs, and a QD monolayer graphene oxide (GO) surface [81]. Nanocomposites (MoS_2_@Au-Au) adsorbed dense 30 nm Au NPs bilayers by coating a thickness-regulated polyethyleneimine intermediate layer (1 nm) on a two-dimensional molybdenum disulfide (MoS_2_) nanosheet, which significantly enhanced the colorimetric capacity and SERS activity. Yu et al. showed that colorimetric signals supported the rapid identification of MPXV, and SERS signals enabled the quantitative detection of MPXV, with LoDs of 0.2 and 0.002 ng/mL (Figure 2d) [71]. Li et al. established a colorimetric and Raman bimodal LFA for the ultrasensitive detection of SARS-CoV-2 N proteins based on 4-mercaptobenzoic acid (MBA) Fe_3_O_4_-Ag^MBA^@Au NPs with magnetic–Raman–colorimetric properties. Under optimal conditions, N protein antibodies could be detected qualitatively in colorimetric mode, with a visual limit of 10^−8^ mg/mL, and quantitatively by SERS signals between 10^−6^ and 10^−10^ mg/mL, with a detection limit of 0.08 pg/mL [82]. Wang et al. developed a colorimetric–fluorescent dual-mode LFA that simultaneously detects SARS-CoV-2-specific IgM and IgG in human serum. This proposal uses SiO_2_@Au@QD nanobeads (NBs) as a label and has high enough sensitivity to require only one microliter of serum sample [83]. Liu et al. developed a colorimetric and fluorescent dual-functional LFA strip that introduced MXene and QDs to simultaneously detect IAV and SARS-CoV-2, which are difficult to distinguish due to their similar symptoms. High-sensitivity multiple detection of 1 ng/mL or 2.4 pg/mL IAV and 1 ng/mL or 6.2 pg/mL SARS-CoV-2 can be completed within 20 min [84]. Liang et al. prepared Ag NPs with ultrathin Au shells (~2 nm) embedded with MBA (Ag^MBA^@Au) and incorporated them into LFAs for colorimetric and SERS bimodal detection of SARS-CoV-2 IgG. Qualitative analysis was performed through visual observation of the T-line, and quantitative analysis was performed by measuring the SERS signal [85]. A detailed list of colorimetric-based LFAs for viral protein detections is presented (Table 1).

## 4. Fluorescence-Based LFAs for Viral Protein Detection

Fluorescence-based LFAs using QDs [68,91,92,93,94,95], fluorescent dye, and dye-doped NPs [96,97,98,99,100] as probes have been developed to improve analysis performance through strong fluorescence intensity [92,101]. For example, LFA, which detects anti-SARV-CoV-2 IgG in serum using lanthanide-doped polystyrene NPs (LNPs), achieves high sensitivity by labeling mouse anti-human IgG antibodies with self-assembled LNPs that act as fluorescent reporters [102]. Recently, for the detection of dengue fever (DF), a new diagnostic platform called spin-enhanced LFA (SELFIA) using fluorescent nanodiamonds (FNDs) as reporters has been reported. Leveraging the inherent magneto-optical properties of negatively charged nitrogen-vacancy centers in FNDs, the SELFIA platform utilizes alternating electromagnetic fields to modulate signals from the fluorescence of FNDs, providing sensitive and specific results. This enabled us to efficiently detect all four dengue non-structural protein (NS1) serotypes (DV1, DV2, DV3, and DV4) [103].

**Figure 3:**
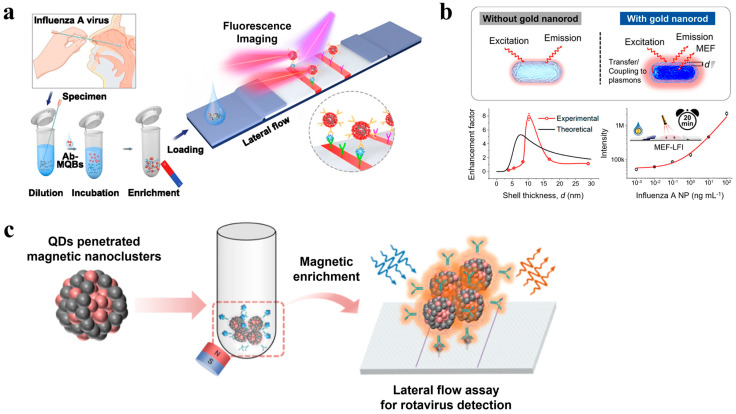
Examples of fluorescence-based LFAs for viral protein detection. (**a**) Scheme of the Magnetic-QD NB-based LFA for the detection of IAV. Reprinted in part with permission from ref. [95]. Copyright 2020 Elsevier. (**b**) Scheme of the mesoporous silica-coated gold nanorod-based LFA for the detection of IAV. Reprinted in part with permission from ref. [104]. Copyright 2023 American Chemical Society. (**c**) Scheme of the magnetic–fluorescent nanocluster composed of Fe_3_O_4_ NPs and CdSe–CdS core–shell QD-based LFA for the detection of rotavirus. Reprinted in part with permission from ref. [105]. Copyright 2023 American Chemical Society.

A metal-enhanced fluorescence (MEF) probe based on core–shell nanostructures using a gold nanorod core, mesoporous silica shell, and cyanine 5 (Cy5) fluorophore has also been designed. In this study, the distance dependence of plasma coupling between Cy5 and gold nanorods was experimentally and theoretically investigated by adjusting the shell thickness to optimize the efficiency of the MEF probe, which significantly improved fluorescence. This platform enabled the high-sensitivity detection of the IAV N protein with an LoD of 0.52 pg/mL within 20 min (Figure 3b) [104].

Although there are a variety of fluorescent reporters, QDs have several prominent features, such as high quantum yields, wide excitation, and narrow, size-adjustable fluorescence emission spectra, which are widely used in fluorescent LFAs [101,106]. Wang et al. developed a magnetic-QD-based dual-mode LFA for the simultaneous high-sensitivity detection of SARS-CoV-2 spoke (S) and N protein antigens. A high-performance magnetic QD with a triple-QD shell (MagTQD) nanotag was used to provide excellent fluorescence signals, enrichment capabilities, and detection potential. The LoDs for the two antigens under direct and enrichment modes were 1 and 0.5 pg/mL, respectively [107]. Li et al. designed an LFA to rapidly detect SARS-CoV-2-specific antibodies using ZnCdSe/ZnS QDs as probes. The LoD of IgG was 48.84 ng/mL [108]. For the multiple detection of four respiratory viruses, a QD NB-based LFA was presented by Chen and their research group. The sandwich complex formed by the detection antibody on the QDs’ surface and the capture antibody on the T-line was excited by ultraviolet light, emitting a red fluorescence signal at a specific wavelength. Their study achieved excellent LoDs of 0.01 ng/mL, 0.05 ng/mL, 0.31 ng/mL, and 0.40 ng/mL for the SARS-CoV-2 antigen, IAV antigen, IBV antigen, and adenovirus (ADV) antigen, respectively [109]. Researchers constructed magnetic–fluorescent CdSe-CdS (QD)/Fe_3_O_4_ nanoclusters (CFNCs) consisting of magnetic NPs and CdSe-CdS core–shells for detecting rotaviruses. QDs are embedded in the inner and outer shells of Fe_3_O_4_ NCs, which can weaken the solubility interactions mediated by solvent polarity, and by increasing the particle-to-particle distances, the CFNCs can overcome the fluorescence quenching effect. The LoD was improved to 1.0 × 10^1^ TCID_50_/mL, and high sensitivity was achieved (Figure 3c) [105]. A detailed list of fluorescence-based LFAs for viral protein detections is tabulated (Table 2).

## 5. SERS-Based LFAs for Viral Protein Detection

**Figure 4:**
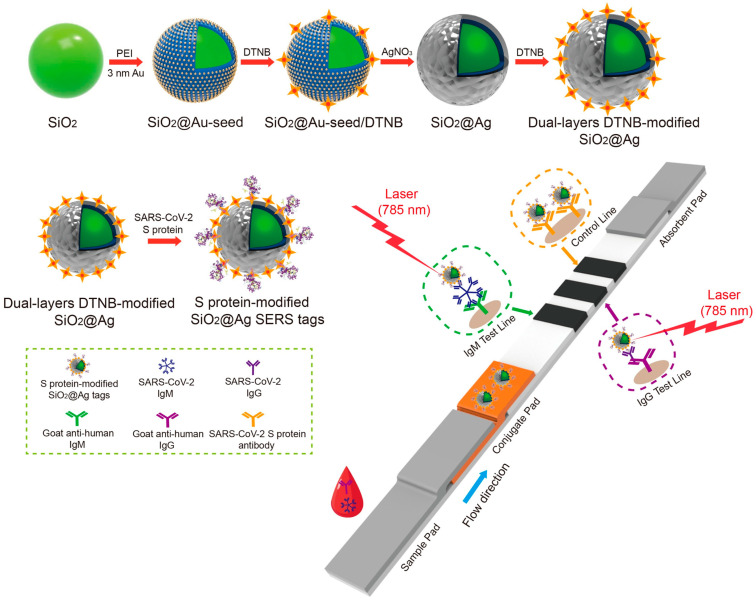
A SERS-based LFA for viral protein detection. Scheme of the dual layer DTNB-modified SiO_2_@Ag SERS tag-based LFA detecting SARS-CoV-2. Reprinted in part with permission from ref. [116]. Copyright 2021 Elsevier.

Based on LSPR excitation, SERS can enhance the Raman signaling of targets adsorbed by Au or Ag metal nanomaterials by several orders of magnitude. For an LFA system using NIR dye-labeled Au NPs as probes, the LoD of SARS-CoV-2-specific IgM/IgG was 100 fg/mL [110]. The LoDs of SARS-CoV-2 and IAV were 5.2 PFU/mL and 23 HAU/mL, respectively, in an LFA platform constructed by attaching malachite green isothiocyanate (MGITC) to Au NPs [111]. However, Raman reporter molecules may dissociate from the surface of Au NPs when exposed to high temperatures, harsh pH, or salty conditions [112]. To address this issue, silica-encapsulated metal NPs have been developed to prevent the desorption of Raman reporter molecules and the adsorption of external species of molecules [113,114]. For this reason, a platform has been reported that can apply silica-encapsulated metal NPs to LFA strips to minimize changes in SERS intensity and maintain stability even under high-temperature conditions (Figure 4) [115,116]. Furthermore, to overcome the limitations of single metals, hybrid fabrication methods for metal cores and metal shells have been developed, such as uniformly coating the Ag core with an ultrathin film of Au to functionalize the surface chemistry of Au and the optical properties of Ag to the probe itself [117,118,119]. This structure enhances the SERS signal via the SERS-enhancing effect of precious metals, large numbers of Raman molecules, and the hotspot effect in the narrow gap between metal cores and metal shells [117]. Metal shells aid in binding to biomolecules, protecting the inner metal core and keeping Raman reporter molecules unaffected by outside conditions, thereby enhancing the biocompatibility and the chemical and signal stability of the particles [85]. Liang et al. developed a SERS/photothermal (PT)-based dual-mode LFA based on Au-core–Ag-shell bimetallic NPs (Au^4-ATP^@Ag NPs) for the antigen detection of infectious disease pathogens. The quantified LoDs for IAV, influenza B virus (IBV), and SARS-CoV-2 were 31.25 pg/mL, 93.75 pg/mL, and 31.25 pg/mL, respectively [119]. Recently, researchers have developed an ultralight SERS probe [120,121,122] by coating an Au core and Ag shell NPs (Au@Ag NPs) with an Au layer and encapsulating Raman molecules between them, in which the Raman reporter between the core and shell has an intense electromagnetic field enhancement effect, which significantly boosts the Raman signals in virus detection [123,124].

Magnetic SERS tags can be used as stable SERS tags for separating target analytes from complex solutions by using external magnetic fields and improving the Raman signal of the target [125,126,127]. LFAs that simultaneously detect IAV H1N1 and human ADV using Fe_3_O_4_@Ag NPs as magnetic SERS nanotags have been reported. A new type of Fe_3_O_4_@Ag magnetic tag combined with a bilayer Raman dye molecule and a target virus-capture antibody allows for the specific recognition of the target virus in solution and SERS detection of the virus via magnetic enrichment and strips. The LoDs of H1N1 and human ADV were 50 pfu/mL and 10 pfu/mL, respectively, making this method 2000 times more sensitive than the standard colloidal Au strip method [128]. For the rapid and accurate measurement of SARS-CoV-2 N proteins, a novel multifunctional NB-based magnetic/fluorescent dual-mode LFA was developed by combining a superparamagnetic Fe_3_O_4_ core with double quantum dot shells (MagDQDs) using polyethyleneimine (PEI) as the carrier shell. Both the magnetic and fluorescent signals showed high sensitivity with LoDs of 0.235 ng/mL and 0.012 ng/mL, respectively [129]. A SERS LFA system based on Fe_3_O_4_@Au magnetic NPs for the simultaneous and supersensitive detection of three respiratory viruses (H1N1/SARS-CoV-2/RSV) has been proposed. 5,5′-Dithiobis-(2-nitrobenzoic acid) (DTNB) was modified from Fe_3_O_4_@Au magnetic NPs to prepare a magnetic SERS tag, providing abundant binding sites for high SERS signals and antibodies. The concentrations of LoD were 85 copies/mL, 8 pg/mL, and 8 pg/mL for H1N1, SARS-CoV-2 and RSV, respectively [130]. A detailed list of SERS-based LFAs for viral protein detection is presented (Table 3).

## 6. Conclusions

This review identified and discussed the principles, mechanisms, and research trends of LFA-based analyses for viral protein detection. Since viral infectious diseases occur and spread worldwide and have a significant influence on human life, accurate and highly sensitive methods for quickly diagnosing infectious diseases and controlling their spread are essential. Thus, we believe that using LFA platforms can be an appropriate method because they have the advantages of significant cost benefits, simple analysis with minimal training or infrastructure, and a short diagnosis time.

Briefly, LFAs can generally be classified based on the detection method. Au NPs are most often used in colorimetric LFAs. The sensitivity is increased through a double detection mode in which peroxidase enzyme activity is used or colorimetric detection is integrated with detection methods such as fluorescence or SERS. For example, fluorescent LFAs using QDs, fluorescent dyes, and dye-doped NPs as probes improve analysis performance through strong fluorescence intensity. Finally, based on the excitation of LSPR, SERS LFAs can improve the Raman signal of the target adsorbed on the precious metal nanostructure by several times. Recently, to maximize SERS intensity and maintain stability even under high-temperature conditions, silica-encapsulated metal NPs and core–shell structures composed of precious metals have been used as probes.

Despite the steady progress of research on LFA systems, developing a simple and stable LFA system capable of exhibiting high selectivity and sensitivity is still a major and fundamental task. Throughout the course of a viral infectious disease, protein levels tend to increase towards the end of the infection, whereas nucleic acid levels are typically higher at the beginning. Based on the literature that we compiled, the idea of integrating the current approaches for the individual detection of proteins and nucleic acids into a unified LFA system capable of simultaneously identifying both components makes it feasible to consistently achieve high-sensitivity detection outcomes throughout the infection period. Additionally, in recent times, the method of multiplexing capabilities for biomarker detection has also actively flourished within the scientific field [132]. In addition to this, trends such as the development of equipment-free signal readout for semi-quantitative and quantitative analysis have been gaining attention due to its indispensable increased usability and accessibility within paper-based sensors, especially the LFA system [133]. As an example, the conventional qualitative detection output of yes/no has been transformed into measurements based on color distance, counts of area color changes, text readout displays, and other technologies [134,135,136]. In detail, this concise review on the trend of transformation was put together based on a review paper by a group of researchers on papers published from 2020 to 2023 [137]. In conclusion, the integration of nanomaterials in LFA systems can be expected to detect viral proteins in clinical diagnosis, and to be used in various fields, such as environmental protection.

## Figures and Tables

**Table 1:** List of colorimetric-based LFAs for viral protein detection.

	Target	Nanoprobe	LoD	Reference
Au NP	HBsAg	Au NP	1.8 ng/mL	[62]
	Nodavirus	Au NP	6 × 10^−3^ TCID_50_	[63]
	SARS-CoV-2	PLASCOP Au NP cluster	0.038 ng/mL	[67]
	Japanese Encephalitis Virus	Au NP	10 pg/mL	[86]
	SARS-CoV-2	Au NP	0.2 mg/mL	[59]
	SARS-CoV-2	Au NP	5 × 10^4^ copies/mL	[65]
Enzyme reaction	IAV, IBV	HRP	0.001~0.00025 HA units (IAV), 0.016~0.004 HA units (IBV)	[73]
	Dengue virus	HRP	5 ng/mL	[74]
	SARS-CoV-2	HRP-labeled Fe_3_O_4_/Au core–shell magnetic NP	400 PFU/mL	[75]
	PVX	ALP-labeled Au NP	0.3 ng/mL	[87]
	SARS-CoV-2	Au@Pd@Pt nanozyme	0.037 ng/mL	[80]
	SARS-CoV-2	ChF/ZnO/CNT nanohybrid	0.05 pg/mL	[88]
	SARS-CoV-2	PDA@MnO_2_ nanocomposite	8.01 pg/mL	[69]
Dual detection	SARS-CoV-2	SiO_2_@Au NP/QD	1 ng/mL, 33 pg/mL (*C, F)	[70]
	IAV	GO-Au/QD-QD	5 × 10^4^ copies/mL, 891 copies/mL (*C, F)	[81]
	MPXV	MoS_2_@Au–Au	0.2 ng/mL, 0.002 ng/mL (*C, S)	[71]
	SARS-CoV-2	Fe_3_O_4_-Ag^MBA^@Au NP	10^−8^ mg/mL, 0.08 pg/mL (*C, S)	[82]
	IAV, SARS-CoV-2	Ti_3_C_2_-QD	1 ng/mL, 2.4 pg/mL (IAV) 1 ng/mL, 6.2 pg/mL (SARS-CoV-2) (*C, F)	[84]
	SARS-CoV-2	Ag^MBA^@Au	10^−6^ mg/mL, 0.22 pg/mL (*C, S)	[85]
	SARS-CoV-2	Ag@Au triangular nanoplate	1 ng/mL, 40 pg/mL (*C, P)	[89]
	SARS-CoV-2	Plasmonic-active Au nanocrown	91.24 pg/mL, 57.21 fg/mL (*C, S)	[90]

*C: colorimetric signal, F: fluorescence signal, S: SERS signal, P: photothermal.

**Table 2:** List of fluorescence-based LFAs for viral protein detection.

	Target	Nanoprobe	LoD	Reference
Fluorophore	SARS-CoV-2	Fluorescent microsphere	100 ng/mL	[98]
	SARS-CoV-2	Aggregation-induced emission_810_ NP	0.236 μg/mL (IgM), 0.125 μg/mL (IgG)	[97]
	Dengue NS1 serotypes (DV1, DV2, DV3, DV4)	FND	0.33, 0.24, 0.10, 1.33 ng/mL	[103]
	IAV	Cy5-mSiO_2_@GNR	0.52 pg /mL	[104]
	SARS-CoV-2	Fluorescent microsphere	0.01 ng/mL	[105]
QD	Zika virus	QD microsphere	0.045 ng/mL	[68]
	Thrombocytopenia syndrome virus	Fluorescent carbon dots (CDs)/SiO_2_ nanosphere	10 pg/mL	[91]
	IAV	CdSe/CdS/ZnS QD	2.5 HAU/mL (H1N1), 0.63 HAU/mL (H3N2)	[93]
	Zika virus	Fluorescent CD-based silica colloid	10 pg/mL	[94]
	IAV	Magnetic-QD NB	22 pfu/mL	[95]
	SARS-CoV-2	MagTQD	0.5 pg/mL	[107]
	SARS-CoV-2, IAV, IBV, ADV	QD NB	0.01, 0.05, 0.31, 0.40 ng/mL	[109]
	Rotavirus	CFNC	1.0 × 10^1^ TCID_50_/mL	[105]

**Table 3:** List of SERS-based LFAs for viral protein detection.

	Target	Nanoprobe	LoD	Reference
Noble metal	Pseudorabies virus	AuAg^4−ATP^@Ag NP	5 ng/mL	[120]
	Avian influenza virus	AuAg^4−ATP^@Ag NP	0.0018 HAU	[121]
	Dengue virus, Zika virus	MGITC-labeled Si-Au NP	1.906 μg/mL	[115]
	SARS-CoV-2	dual-layer DTNB-modified SiO_2_@Ag NP	1 pg/mL	[116]
	Rotavirus	Au/DTNB/Ag/DTNB	8 pg/mL	[117]
	West Nile virus	Au@Ag NP	0.2 × 10^2^ copy/μL	[118]
	SARS-CoV-2	NIR-797-ITC-labeled Au nanostar	100 fg/mL	[110]
	SARS-CoV-2, IAV	MGITC-labeled Au NP	5.2 PFU/mL (SARS-CoV-2), 23 HAU/mL (IAV)	[111]
Magnetic NP	IAV, human ADV	Fe_3_O_4_@Ag magnetic tag	50 pfu/mL, 10 pfu/mL	[128]
	SARS-CoV-2	MagDQD	0.235 ng/mL, 0.012 ng/mL (*M, F)	[129]
	IAV, SARS-CoV-2, respiratory syncytial virus	Fe_3_O_4_/DTNB@Au/DTNB	85 copies/mL, 8 pg/mL 8 pg/mL	[130]
	SARS-CoV-2	DTNB–encoded satellite Fe_3_O_4_@Au SERS tag	23 pg/mL, 2 pg/mL	[131]

*M: magnetic signal, F: fluorescence signal.

## Data Availability

Not applicable.
